# Catalogue of the Preparations Illustrative of Normal, Abnormal, and Morbid Structure, Human and Comparative, Constituting the Anatomical Museum of George Langstaff, &c. &c.

**Published:** 1843-10-01

**Authors:** 


### CATALOGUE OF THE PREPARATIONS ILLUSTRATIVE OF NORMAL, ABNORMAL, AND MORBID STRUCTURE, HUMAN AND COMPARATIVE, CONSTITUTING THE ANATOMICAL MUSEUM OF *George Langstaff*, &c. &c. London, Churchill. 1842.

IT was, we believe, old Dr. Gregory of Edinburgh, who used so earnestly to impress on the minds of his pupils the vast importance of assiduously perusing Morgagni's celebrated work *De Sedibus et Causis Morborum* in the terms, *Nocturnâ versate manu, versate diurnâ*. The habit of connecting the morbid appearances found after death with the symptoms observed during life, and of placing them, as it were, in juxta-position, being the very best, nay in fact the only means of preparing and qualifying the medical practitioner for attaining and forming a correct diagnosis at the bed-side of his patient. To point out the value and importance of studying morbid structure and of tracing it through all its stages and gradations from the first blush of capillary redness to the final disorganisation of parts, would at the present day be a work of supererogation. That the thing is fully recognized, the numerous Anatomical Museums of Morbid Structure throughout the metropolis evince. The opportunities of studying this important branch of pathology in London, containing as it does, the Hunterian Museum, that stupendous monument of human industry, together with the numerous other Museums attached to the various hospitals, and contributed to by the hands of such men as Cooper, Brodie, Lawrence, &c. &c. are to be surpassed nowhere. The collection of the specimens of morbid structure, of which we now present the catalogue to the notice of our readers, was commenced some thirty years ago, at a time, says the author, when the study of morbid anatomy was but little practised, and almost every post-mortem examination offered subject of interesting and novel investigation. In forming his collection, Mr. Langstaff was assisted by several of his medical friends, to many of whom he here expresses his acknowledgments. The preparations are intended to illustrate not only morbid, but normal and abnormal structure. The arrangement is physiological—he commences with the Osseous System, and gives cases of some of the principal diseases of the bones. Then the Vascular System, under which he gives the Normal, Abnormal, and Morbid Anatomy of the Pericardium and Heart, and of the Arteries and Veins—then the Nervous System, including the *Brain* and *Spinal Cord*, with their membranes, and the nerves. Next the *Organs of Respiration*—*Organs of Digestion*—*Urinary Organs*—the male and female generative organs. Then, under the head of Miscellaneous Preparations, we have two Chapters, containing the descriptions of some matters which should have come in before, but had escaped notice. Several specimens of *Comparative Anatomy* find a place in Mr. Langstaff's Catalogue.

We shall present our readers with some extracts from the most interesting parts of this work—and shall commence with the *Vascular System*, there being nothing very striking or novel in the Chapters descriptive of the Osseous System. In extracting the cases we shall retain the numbers affixed to the originals.

### *Malformation of the Heart.*

282. In the heart of a boy eight years old the cavities were found to be very large, and the muscular structure very firm. Nearly three ounces of water found in the pericardium—foramen ovale open—valvula mitralis shortened and thickened; the Eustachian valve extensive and reticulated. The aorta and its vessels were very large; the sigmoid valves of this vessel and of the pulmonary artery thickened. From birth he had all the signs of hydrocephalus, with symptoms denoting malformation of the heart; his lips of a purple color, skin rather cold; pulse full and irregular; these signs were attended with dyspnœa. He was attacked with pertussis, convulsions came on, and he expired in one of the paroxysms. The head was immensely large; the arachnoid membrane and pia mater were much thickened, and a milky fluid was effused between them; the vessels of the brain were surcharged with blood, and the structure of the cerebrum was very dense; the septum lucidum and fornix softened—four ounces of fluid in the lateral ventricles, and the third ventricle was filled with water which produced pressure on the optic nerves.

286. Here we have the heart of a boy ten years of age. From birth he had symptoms denoting mal-formation of the heart; he was a complete cæruleus; pulse generally very full and irregular. About a fortnight before death he had a severe attack of scarlatina; purpura hæmorrhagica nearly all over his body; this was followed by hydrothorax and ascites—in both sides of the chest there was a considerable quantity of serous effusion—lungs œdematous and partly hepatised—four ounces of fluid in pericardium—heart remarkably large, and presented a very irregular appearance; its apex very obtuse.

The parietes of the left ventricle were thick, the cavity capacious, the mitral valve and lining of the auricle much thickened. At the superior and central part of the septum ventriculorum, there is an aperture about the size of a shilling, allowing a free communication between the ventricles. Two of the aortic valves adhere very firmly at their superior part—they are slightly thickened. The aorta is very large as are also the coronary arteries—foramen ovale open—fossa ovalis deep—the ductus arteriosus closed—parietes of right ventricle thick—cavity large—the tricuspid valve and lining of the auricle thickened; pulmonary artery large.

This is a remarkable specimen of mal-formation of the heart—it is surprising the patient lived so long.

*Morbid Anatomy of the Pericardium and Heart.*

294. Here is a specimen displaying pericarditis. The patient, a gentleman 46 years of age, had lived intemperately. For several years he had been afflicted with symptoms denoting a diseased liver; about twelve months previous to his death he was troubled with a dry cough; dyspnœa, and symptoms of organic disease of the heart supervened, which gradually increased in violence—he died of effusion into the chest. The liver was found to be enormously large, of a deep yellow colour—the gall-bladder was much distended with viscid bile—spleen very large and extremely soft. In the right side of the chest were found three pints of sanious fluid—the lobes of the lung were firmly agglutinated by organized lymph and much flattened—the pleura costalis thickly coated with lymph.—The lung on the left side healthy—four ounces of yellowish fluid in the pericardium—its internal surface and that reflected over the heart highly inflamed, and lymph deposited which was organized; firm adhesions had been effected, more especially on the left side of the heart near the auricle and the root of the pulmonary artery—the left auricle and ventricle very capacious, the parietes of the latter rather thin—mitral valve normal, except a few opaque spots near its origin—the right side of the heart normal. This our author very justly pronounces to be a very valuable preparation—we think its value would have been enhanced considerably, had the history of the case been given more fully—one circumstance struck us as rather strange, viz. the thinness of the parietes of the left ventricle—from the pericardial adhesions found on the left side of the heart one would have expected thickening of the parietes of this ventricle. We have no symptoms which can at all account for, or at least which can be at all referred to, the inflammatory appearances found in the lungs.

In 297 we have another instance of disease of the pericardium and of the heart itself in a man 45 years old and of intemperate habits, who, for several years before any appearance of heart disease, suffered from an affection of the liver. The symptoms of organic disease of the heart came on gradually, at length became exceedingly severe and caused hydrothorax. The pulsation of the heart was violent and extensive and generally irregular; there was also distressing dyspnœa, great anxiety and irritability, with a bluish suffused countenance. A considerable quantity of water was found in each side of the chest. The pericardium adhered very firmly to the reflected portion by lymph, which was in great quantity, and organized—the right auricle very capacious, and its internal surface inflamed—ventricle healthy. The left auricle large—its internal lining inflamed and partly ulcerated—the mitral valve very much thickened—ventricle immensely capacious, the parietes natural.

An immense quantity of water in the abdomen; liver very large.

299. Here we have the heart affected with chronic pericarditis according to our author's mode of stating the case. The patient, 19 years of age, tall, delicately formed, had for nearly four years evinced signs of phthisis pulmonalis. About six months previous to his death, symptoms of pneumonia came on, accompanied with great difficulty in breathing; these symptoms gradually increased, and he was occasionally attacked with syncope. Soon after this period the heart became affected—he was annoyed with violent palpitation. The expectoration was muco-purulent, but never profuse; hectic fever supervened; but, what is very singular in this case, the emaciation of the body was not so great as we generally notice, where phthisis has run through its different stages.

About a fortnight previous to his death, the mucous surface of the trachea and intestines became diseased; he lost his voice, and had symptoms similar to those in cynanche trachealis, and colliquative diarrhœa.

The adhesions of the pleuræ were extremely firm—lungs filled with tubercles and vomicæ of different sizes; the bronchial glands were similarly affected, and compressed the bronchi: the lining of the trachea was inflamed and the bronchi slightly ulcerated. The pericardium, extremely dense in structure, was distended with a great quantity of fluid mixed with large flakes of coagulable lymph. The internal surface of this membrane was thickly coated with organized lymph. In the liver was found a great number of scrofulous tubercles, and many of the mesenteric glands were converted into caseous matter—mucous glands of the ileum and cæcum enlarged, some of them ulcerated.

301. Here we have a portion of the left ventricle of the heart of a man, 60 years of age, shewing the effects of chronic pericarditis. He had for several years been afflicted with gout; during the last three years of his life, he had cough and dyspnœa, with palpitation of the heart. About a fortnight before his death, he was attacked with a very severe paroxysm of gout, succeeded by metastasis to the heart, and he expired suddenly. The pleuræ costalis and pulmonalis, adhered very firmly on both sides of the thorax; sections of the lungs shewed the effects of sero-purulent infiltration. The pericardium contained a considerable quantity of yellow serum mixed with flakes of very white coagulated lymph; the internal surface was thickly coated with organized lymph, which adhered very firmly, proving the effects of chronic inflammation; and a dense adventitious membrane was formed over the reflected portion of the pericardium; the lymph was remarkably white, and, in many parts, superficially granulated. Our author has before noticed this singular appearance of the coagulated lymph in pericarditis, the effect of gout, so very different from what we see in acute pericarditis in other circumstances.

304. A case in which the heart and lungs were inflamed. A boy, 12 years of age, very delicate, was attacked with cynanche trachealis; symptoms were very violent. He died on the sixth day from the commencement of the disease.

Pleuræ inflamed very much—lungs hepatized—they also contained a great number of tubercles—several also beneath the pleuræ. The bronchial glands were converted into fleshy tumors, which were very vascular and compressed the bronchi—there was also a small vascular tumor in one of the pulmonary veins, which adhered very firmly. The pericardium contained about an ounce of water—its internal surface was inflamed, as were also the muscular structure of the heart, the linings of the ventricles and auricles and the coats of the arteries. The inner surface of the trachea and bronchi was highly inflamed, but lymph had not been deposited.

305. Here we have a portion of the left ventricle of the heart, shewing two small lacerated openings near its apex. A man, 47 years of age, very robust and plethoric, experienced a very severe attack of acute rheumatism in the hands and feet, which was followed by metastasis to the heart, and caused violent palpitation, accompanied with excessive pain in the chest, and dyspnœa. These symptoms increased in violence, and he expired suddenly, on the fourth day from the commencement of the attack.

Pleuræ and lungs inflamed. On opening the pericardium, there was found a large quantity of fluid blood, which had escaped from the cavity of the heart, by two small lacerations near its apex. The heart was natural in size, but the serous covering, the lining of the auricles and ventricles, the muscular structure, and the internal coat of the aorta and pulmonary artery, were inflamed.

307. Heart of a lady, 63 years of age, of a very corpulent habit—had been subject to palpitation of the heart during six years, accompanied with violent headache. She frequently complained of pulsation and a singing noise in her ears; her sight diminished in power; pupils contracted; she was very nervous, and subject to attacks of syncope; the lips mostly of a purple colour. About twelve months before her death the palpitation of the heart became more troublesome; the pulsation and noise in the ears increased, and her mental powers became affected. She died suddenly. The vessels of the scalp and pericranium, as well as those of the cranium, were extremely turgid with blood; the arachnoid membrane lining the dura mater was inflamed, and slightly thickened by lymph, and that portion covering the pia mater was thickened by chronic inflammation. A considerable quantity of water mixed with coagulated blood was found between the pia mater and the external surface of the brain—there was no lesion of the substance of the brain.

The membranes covering the base of the brain were much thickened, and blood had been effused between the pia mater and the cerebrum, and between the latter and the cerebellum. This hæmorrhage was discovered to have been occasioned by the rupture of an aneurysm in the anterior-superior part of the basilar artery, where it gives off the posterior cerebral arteries; it had lacerated the membranes at the posterior part of the infundibulum, and thus the blood was forced into the third, lateral, and fourth ventricles, the fornix and septum lucidum having been broken down by the force of the blood. The carotids and anterior cerebral arteries were aneurysmal, and on the right side an aneurysmal sac would shortly have burst. Almost all the arteries at the base of the brain were in a morbid state. Two ounces of fluid in the pericardium—heart large—between its serous covering and muscular structure a great deal of fat—auricles and ventricles very large—left ventricle very large and its parietes very thickened—mitral valve thickened—edges of tricuspid valve partly cartilaginous. The aorta and vessels given off from its arch remarkably large, particularly the arteria innominata; the internal coat of these arteries thickened with large patches of lymph. Lungs very much loaded with blood and mucus. The liver was very large and its serous covering thickly coated with organized lymph: substance of the liver as dense as carcinoma—it was nodulated.

308. Heart of a man 74 years of age. He had been distressed with difficulty of breathing for several years, as also palpitation of the heart, which symptoms had increased in violence for six months previous to his death. The pulsation of the heart and arteries very powerful and invariably irregular, his lips of a purple colour; he had all the symptoms denoting angina pectoris very strongly marked.

His breathing was very difficult—his sleep interrupted by sudden startings, and frightful dreams, secretion of urine scanty—legs became œdematous, and he expired suddenly. Ten ounces of serous fluid were found in the pericardium, and four pints of the same kind of fluid in the left cavity of the chest, and two and a half in the right. Heart enormously large, from the size of its auricles and its left ventricle, the parietes of which were very thin and soft. The semilunar valves of the aorta very much thickened, and shortened by cartilaginous and osseous deposits, and by organized lymph; two of them were lacerated, which must have allowed of regurgitation of the blood into the ventricle. It is very remarkable that there were three coronary arteries, and they were not diseased, which, our author says, satisfactorily shows that angina pectoris is not always caused by ossification, or any other morbid state of these vessels.

309. The left ventricle of the heart of a man, 73 years of age. He had been troubled with asthma and palpitation for several years; during the last eight months of his life the symptoms became more serious, and signs of hydrothorax ensued. The pulse was generally intermittent, the breathing laborious, and he was occasionally attacked with paroxysms of dyspnœa, which threatened complete suspension of respiration. The *heart's action was very powerful,* and its could be felt extensively on the chest, and nearly as low as the umbilical region.

His legs and thighs became œdematous, action of the heart more distressing, and he died suddenly.

The pericardium contained six ounces of water; left ventricle very capacious, and *its parietes very thin*—muscular structure very flaccid (passive aneurysm of Corvisart). We beg to call the reader's attention to the words in italics. The great extent and power of the heart's action does not appear very reconcileable with the great attenuation of the left ventricle, and the normal condition of the right; for it should have been stated that the right side of the heart was normal.

312. Heart of a gentleman, 55 years of age. For several years he had evinced symptoms of hepatic disease—countenance sallow—conjunctivæ yellow—bowels generally constipated—he was dyspeptic. Next came symptoms of organic disease of the heart, which increased in violence—breathing laborious, attended with great anxiety, and sometimes followed by a state of syncope—in one of these attacks he expired. The pulse had always been so frequent and irregular as not to be countable.

In the pericardium there was half a pint of bloody-looking fluid; on the inner surface of this membrane, and on its reflected portion, lymph was thickly deposited, and adhered firmly. The muscular structure of both ventricles very soft. The left ventricle was very capacious and its parietes thick—mitral valve thickened, but the opening not constricted. Cavity of right ventricle large, but the parietes thin.

Semilunar valves of aorta were ossified, particularly at their base, which may account for the irregularity of the heart's action as indicated by the pulse—there was much water tinged with blood in both sides of the chest.

*Morbid Anatomy of Arteries and Veins.*

393. Portions of the thoracic aorta showing inflammation and ulceration of the internal coat, from a man 34 years of age, who had been afflicted with organic disease of the heart nearly two years. Effusion of fluid into the thorax and abdomen came on a short time before his death; there was considerable hæmorrhage from the lungs and intestines, and the integuments of most parts of his body were affected with purpura. Heart very large—left auricle and ventricle very capacious—the parietes of the latter very thick—mitral valve healthy. The aortic valves slightly shortened and thickened and also inflamed, and one of the coronary arteries completely obliterated by lymph. The entire of the aorta inflamed, and some portions of the internal coat ulcerated. Signs of purpura hæmorrhagica beneath the pleura pulmonalis—lungs congested by sero-purulent infiltration—all the viscera of the abdomen surcharged with blood; the serous coats affected with purpura, and there was a considerable quantity of coagulated blood in the intestines—a great collection of fluid, on both sides of the thorax, in the pericardium and abdomen.

" This," says our author," was a case of arteritis, excited by the magnitude and power of the heart's action, which probably might have been subdued by depletion and digitalis, if the heart had not been affected."

436. Aorta immensely dilated, particularly at its origin, arch, and descending portion.

The sigmoid valves and coronary arteries slightly ossified—the internal coat of the aorta, and vessels of the arch greatly thickened by dense cartilage and bone. The man, aged 65 years, for more than twelve months had symptoms denoting aneurysm of the aorta; effusion into the chest supervened, and he expired suddenly.

There was a considerable quantity of serum in both cavities of the chest; the lungs were emphysematous, and some of the lobes were infiltrated with sero-purulent fluid. The pericardium contained nearly six ounces of water; heart very large; parietes of the left ventricle very thick; the cavity was very capacious, and the aperture of the mitral valve semi-cartilaginous.

438. Two portions of the thoracic aorta, showing inflammation and partial ulceration of the internal coat, with considerable thickening by cartilaginous and osseous deposits. One of the coronary arteries obliterated by lymph, whilst the aperture of the other only admitted a small bristle. The patient, 34 years of age, had been affected with cough and distressing dyspnœa for nearly two years, also violent palpitation of the heart; pulse full and very irregular; lips purple. Symptoms of hydrothorax came on, succeeded by ascites, and, about a fortnight previous to his dissolution, symptoms of purpura hæmorrhagica appeared on most parts of his body; he had also had hæmoptysis and intestinal hæmorrhage, and he died suddenly. Heart tremendously large; left auricle and ventricle very capacious; parietes of the latter very thick; aorta and pulmonary artery highly inflamed; pericardium contained about four ounces of water. About two pints of water in both sides of the chest.

446. Inferior part of the abdominal aorta, the iliacs, and main arteries of the thigh and leg ossified; some of them entirely obliterated. A man 77 years of age, who had enjoyed excellent health during the greatest part of his life; about three years previous to death suffered several attacks of erysipelas in the right leg and foot. At length the toes became extremely painful, and disturbed his rest in the night; they next began to show want of vitality, and the integuments of the foot and leg became cold and discoloured; no pulsation could be felt in the anterior or posterior tibial arteries, and sphacelation supervened. The mortification proceeded very slowly to the plantar fascia, and destroyed the integuments of the sole of the foot—the discharge of pus very offensive. Fever came on, affected the brain, and terminated his life.

A considerable quantity of fluid in the pericardium; muscular structure of the heart soft. Osseous matter on the chordæ tendineæ of the mitral valve; coronary arteries much diminished in calibre by bony matter, and some of the branches were obliterated. He never had had any symptoms of angina pectoris.

The internal coat of the thoracic and abdominal aorta was ossified in irregular dense plates. Iliacæ communes partly bony and cartilaginous, and firm portions of coagulated blood were found in them, as in aneurysmal sacs. A portion of bone extended from the iliaca externa to the iliaca interna, and the latter was completely obliterated by solid bone for nearly an inch. Not much disease in the femoral artery, till about an inch below, where it gave off the profunda, but from this to the ham it was a complete bony tube; the popliteal artery was obliterated by bone and coagulated blood, and the anterior and posterior tibial arteries were obliterated.

456. The external iliac artery and vein, also the femoral artery and vein; from a man 70 years of age. He had enjoyed good health for many years, although he was troubled with a large foul ulcer with thickened edges on the middle of the leg near the tibia; the veins were varicose. At length the wound became very painful, the pain daily increased, and there was an ichorous discharge: the surface of the sore assumed a dark hue, and the temperature of the foot and leg was considerably diminished; in the course of a week they showed all the signs of gangrene. He suffered agonizing pain—health became disordered—integuments of foot and leg began to slough, and the discharge of pus was very offensive. He wished very much to have the leg amputated, contrary to the wishes of his surgeon—his wish was ultimately complied with, and the leg was taken off above the knee. The stump healed in the course of ten days.

*Examination of the Limb.*—Integuments hard and dry; muscles dark-coloured, and extremely putrid; periosteum dense and separated from tibia by pus. About seven weeks after the operation he was suddenly seized with violent palpitation of the heart and dyspnœa, somewhat resembling angina pectoris, and lived only 24 hours.

*Inspectio Cadaveris.*—More than a pint of water in each side of the chest; old adhesions of the pleuræ; lungs emphysematous; bronchial tubes surcharged with a dark-coloured secretion, and in parts the air-cells were enormously enlarged: pericardium contained about four ounces of water; heart healthy in its structure; valves of the aorta shortened and thickened by cartilaginous deposits; coronary arteries diminished in calibre by osseous matter; spleen converted into a cartilaginous mass. A portion of the tendinous structure of the diaphragm ossified. The main arteries of the thigh, and to where the external iliac is given off, were obliterated by lymph and coagulated blood. Likewise the veins belonging to the thigh, and as high as the external iliac, where it assists in forming the vena cava inferior, were shrivelled and obliterated.

461. Arch of the aorta, with a portion of the trachea, bronchi, and bronchial glands. Internal coat of the aorta is slightly thickened by lymph; and at the anterior part of the aorta, and nearly opposite the origin of the left subclavian artery, there is an aneurysm; its aperture large, but the sac very small. What is very remarkable in this preparation is, that by adhesive inflammation of the cellular tissue belonging to the bronchial glands, and the external coat of the inferior part of the aorta, a firm bond of union has been effected, and covered the sac of the aneurysm, which no doubt prevented the increase of the tumor. This our author considers an effort of Nature to effect a spontaneous cure. The patient, a woman 23 years of age, died of phthisis pulmonalis. The lungs were filled with miliary tubercles and vomicæ.

464. An aneurysm in the arch of the aorta, of considerable magnitude; it had ascended as high as the inferior portion of the thyroid gland; firm adhesion had taken place between the posterior part of the sac of the aneurysm and the anterior surface of the trachea; and an ulcerated opening of communication had been produced between the aneurysm and the trachea; the patient died suddenly from hæmorrhage. The parietes of the aneurysm not thicker than those of a healthy aorta. The sac contains a very dense lamellated coagulum, mixed with fibrine—internal coat of the aorta much inflamed—the left carotid nearly obliterated. This specimen is particularly valuable for showing how nearly a spontaneous cure had been effected.

467. An immense aneurysm at the arch of the aorta, which produced partial absorption of a portion of the sternum and some of the ribs. The patient was a woman, 37 years of age—the disease had proceeded gradually for 16 months. About a fortnight before her death, the integuments covering the enlargement became inflamed, sphacelation ensued, and the aneurysm burst; the hæmorrhage was tremendous. The sac of the aneurysm had caused absorption of a considerable portion of the sternum, and the remaining part was carious, as also some of the ribs. The aorta, from its origin as far as the iliac arteries, was greatly dilated; the internal coat converted into solid bone and cartilage, and in some parts ulceration had taken place. Heart large—parietes of left ventricle very thick, and cavity large.

It is remarkable that the inner coat of the aorta should have become ossified to such a degree in so young a person.

470. An aneurysm at the descending portion of the aorta, which burst into the œsophagus and the right bronchus. The patient, 60 years of age, complained of having suffered greatly from the effects of a cough and pain in the chest, accompanied with great difficulty in breathing; his face looked sallow, lips of a purple colour, and he appeared greatly dejected; pulse 120, small, but not irregular; he also said he experienced great trouble in swallowing for some time.

Fauces appeared healthy—colour of lips and skin denoted disease of heart and liver—he was seized with hæmatemesis, which was so profuse as to threaten death—this, however, gradually subsided, but he discharged blood with his fæces—in three days after the hæmatemesis recurred, accompanied by hæmoptysis, which caused immediate suffocation.

Lungs of the right side partly hepatised, and one of the lobes of the left cavity was injected with blood. At the descending part of the aorta, near the origin of the left subclavian artery and the under surface of its arch, there is an aneurysm of moderate size: the sac is partly filled with coagulated lymph and blood; firm adhesions had been formed between it, part of the left bronchus and the œsophagus, and from ulcerative absorption of the parts, the aneurysm burst into those tubes. The aperture of the aneurysm is large for the size of the sac. Liver large, structure dense and granulated. Much blood in the intestines.

The author remarks it as very singular in this case, that there were no diagnostic symptoms to denote the existence of an aneurysm—and yet we think that the absence of signs in the lungs which could account for the cough, pain in the chest, and the great difficulty in breathing, as also the purple colour of the lips, might have induced one to suspect either the heart, or some of its great vessels, as being the seat of the mischief; and when to this we add the difficulty of swallowing, we do think that there was nearly enough to bring the disease home to its right place, viz. to some part of the aorta contiguous to the œsophagus.

477. An aneurysm of the descending portion of the aorta, the sac ulcerated. A man, 30 years of age, of a very plethoric temperament, had been troubled with palpitation of the heart for nearly eight months, accompanied frequently with violent pain in the head, and dyspnœa. Some medical men referred his disease to the heart, some to the digestive organs.

For about three months previous to his death he was annoyed with cough and difficulty of breathing: he became very irritable, and expired suddenly.

Brain natural—blood-vessels empty—much coagulated arterial blood in both cavities of the thorax—heart and lungs healthy, but exsanguine. An aneurysm was detected in the descending portion of the aorta about two inches below the point where it gives off the subclavian artery, and the sac had ulcerated.

The lesion was found to have been produced by a large spiculum of bone, which had its origin from the anterior surface of the body of the third dorsal vertebra, and had occasioned ulceration of portions of the pleuræ, which allowed the blood to flow from the aneurysm into both cavities of the thorax.

*Brain and Spinal Cord.—Morbid Anatomy.*

501. A portion of brain, and its tunics. From a man, 30 years of age, a confirmed drunkard; he was seized with delirium tremens, which was succeeded by symptoms of typhus fever; he became furiously delirious, and died in about a week from the commencement of the attack.

*Inspectio.*—Great effusion of blood between the arachnoid and pia mater, partly fluid and partly coagulated; these tunics were highly inflamed, as was the substance of the brain—there was likewise a considerable quantity of fluid in the ventricles. All the vessels of the brain were much distended. Liver much enlarged—structure granulated, as also that of the kidneys.

504. A portion of cerebellum, with the arachnoid membrane and pia mater. The arachnoid membrane and pia mater very much inflamed, as also the substance of the brain.

From a very plethoric man, who was attacked with ear-ache on the left side, which was very distressing—the symptoms increased in violence for three days, when a profuse discharge of pus escaped from the ear.

The patient imagined the complaint to be merely the effect of a severe cold, and employed only fomentations and poultices. On the fourth day, symptoms of apoplexy appeared, and the author was called in—he was quite insensible—breathing stertorous—pulse full and hard; and the vessels of the conjunctivæ surcharged. He was bled largely from the arm—a blister was applied to the nape of the neck, an active purgative given and cold water applied to the head—he died in twelve hours.

*Inspectio.*—On cutting open the dura mater on the left side, a considerable quantity of pus escaped from between the arachnoid membranes; and they as well as the pia mater were highly inflamed and thickened—the substance of the brain was very firm, and inflamed; and there was pus in the ventricles—pus was also found in the semicircular canals, cochlea and vestibulum, and the lining of the meatus auditorius externus was in a sphacelated state.

538. A portion of dura mater and arachnoid membrane, with a tumor in the plexus choroides.

From a man 64 years of age, who had a malignant tumor in the scalp at the vertex of the head; which caused caries in a portion of the parietal bone. The bone was absorbed, the dura mater exposed, and the pulsation of the arteries of the brain was seen. It was expected that the longitudinal sinus would be opened by ulceration, this, however, did not take place; the surface of the dura mater assumed a healthy appearance; granulations formed, and there was a considerable discharge of pus. The man continued in this state for two years without experiencing much uneasiness, except what he called "slight pain in the head." He died of serous apoplexy.

*Inspectio.*—Longitudinal sinus inflamed; the arachnoid membrane lining the dura mater, and covering the pia mater, was thickened by chronic inflammation; the pia mater was also thickened, and there was a considerable quantity of water beneath this tunic. The ventricles contained about six ounces of water, and in each plexus choroides there was a tumor, the size of a horse-bean.

543. Dura mater, with the other tunics, and a portion of the cerebrum. Blood effused between arachnoid and pia mater, which adheres firmly to the former—the arachnoid covering the pia mater is thickened. A plethoric man, 67 years of age, was thrown out of a gig, and fell on the right side of his body—he was taken up insensible. When seen by the medical attendant, he appeared labouring under compression of the brain; breathing stertorous, pupils contracted; vessels of the conjunctivæ much distended—pulse full and hard.

On examining the head to ascertain whether there was fracture of the skull, there was detected a considerable enlargement on the right side of the head beneath the integuments covering the temporal and parietal bones—no signs of fracture in any part of the cranium—some of the true ribs were fractured—he was bled, and purged—head shaved, and a cold evaporating lotion applied—on the next day he was able to answer questions, but had no recollection of the accident; the pulse remaining full and hard, he was bled again—leeches to head, &c.; he still complained of pain in the head—the brain became very much affected, and convulsion came on—he lived fourteen days from the time of the accident.

*Inspectio.*—Water was found between the arachnoid and pia mater—on the left side a considerable quantity of coagulated blood was found, firmly adhering to the arachnoid membrane lining the dura mater—the brain itself was inflamed.

From the Section containing the Morbid Anatomy of the Organs of Respiration we decline making any extracts, the pathology of these organs being so very much improved since the time Mr. Langstaff commenced his collection—and shall proceed to the *Organs of Digestion*—commencing with the *Œsophagus.*

872. In this preparation we have the larynx, pharynx, trachea, and œsophagus. From a woman 50 years of age, who had been long suffering from stricture in the œsophagus. She was much emaciated. A surgeon, in endeavouring to pass a bougie through the strictured part, employed considerable force, so that the patient seemed half suffocated and made efforts to vomit. Two days after the attempt she died.

*Inspection.*—The stricture was found to be situated about two inches below the posterior portion of the cricoid cartilage; it was very narrow and nearly two inches and a half in length. The disease was cancer, which had commenced in the cellular tissue, between the coats of the œsophagus, and by progressive absorption had made its way through the muscular and mucous coats into the tube of the œsophagus. A little below the superior aperture of the stricture the posterior part of the œsophagus was lacerated to as far as the inferior opening. The internal lining of the larynx and trachea was slightly thickened.

873. Base of tongue, pharynx, larynx, trachea and œsophagus. A woman 56 years of age, who had experienced great difficulty in swallowing for several years; a few months previous to her death she could only swallow fluids in small quantity, and that with the greatest difficulty. She died of inanition.

There was a stricture in the œsophagus, nearly an inch in length, the constriction only admitting a piece of glass not much larger than a probe. The stricture is nearly at the commencement of the œsophagus; it is formed in the cellular tissue, and had caused partial absorption of the muscular and mucous coats in the constricted part.

896. Here we have the inferior part of a stomach affected with carcinoma. The aperture of the pylorus greatly constricted by the growth of carcinomatous tubercles between its coats—there is an ulcerated opening through all the tunics, to the extent of half-a-crown, near the pylorus: the edges appear to have healed, and had united to the peritoneal coat of the pancreas which had prevented the contents of the stomach from escaping into the abdomen:—at about an inch and a half from the opening near the pylorus there is another ulcer about the size of a sixpence, the edges well defined; this only destroyed the mucous and muscular coats. There were several cancerous tubercles on the peritoneal covering of the stomach and pancreas; the latter was healthy—cancerous tumours also in the liver.

The patient, a woman 59 years of age, had been for nearly two years afflicted with an affection of the stomach, accompanied with symptoms denoting diseased liver. Several months before death she could scarcely keep anything on her stomach, and the bowels were generally costive.

897. A portion of stomach and a medullary tumor in the liver. A man, 50 years old, a baker, had for several years been subject to a disordered state of the stomach and intestines.

For nearly a year and a half previous to his death his health declined, he complained of pain and burning heat in his stomach, and sour eructations, and it was with great difficulty he could retain anything in his stomach—he became much emaciated and died.

*Inspection.*—Liver immensely large—many medullary tubera beneath its serous surface, as also in its substance.

Nearly the whole of the small arch of the stomach was affected with various sized medullary tumors situated between the different coats, and they were all much thickened, especially the mucous and serous, and the tumors were seen beneath them. Most of the mucous glands of the ileum and cæcum were enlarged.

For the same reason that we declined making any extracts from the section containing the Morbid Anatomy of the Respiratory Organs, we must also decline making any from that which presents the Morbid

Anatomy of the *Urinary Organs.* The vast improvements introduced within the last few years into the pathology of these organs by the labours of Prout, Rayer, Bright and Brodie, have given an entirely new aspect to this important branch of medicine.

We now take leave of our author, but not without expressing our high opinion of the ardent zeal and unwearied industry which must have sustained and borne him on in his very arduous and praise-worthy task. He would however have considerably enhanced the value of his contribution to pathological science, had he been more minute and more precise in his details of the symptoms during life. There is not that precision, that consecutiveness in the histories of the several cases, which modern pathologists look for. With respect to the imperfection now so glaring in the details of the cases under the head of the Morbid Anatomy of the Organs of Respiration, such imperfection must be charged less to our author than to the times in which he made his collection, times by the way, when cough, dyspnœa, and pain in the chest, contained almost all that was known of thoracic pathology.

